# Does public service motivation matter in Moroccan public hospitals? A multiple embedded case study

**DOI:** 10.1186/s12939-019-1053-8

**Published:** 2019-10-22

**Authors:** Zakaria Belrhiti, Wim Van Damme, Abdelmounim Belalia, Bruno Marchal

**Affiliations:** 1 National School of Public Health, Rabat, Morocco; 2 Department of Public Health, Institute of Tropical Medicine, Antwerp, Belgium; 3 Vrije Universiteit Brussel, Brussels, Belgium

**Keywords:** Public service motivation, Health workers, Motivation, Morocco, Intrinsic motivation, Extrinsic motivation, Hospitals

## Abstract

**Background:**

The motivation of health workers is a key concern of policy makers, practitioners and researchers. Public Service Motivation (PSM), defined as the altruistic desire to serve the common interest, to serve others and to help patients and their families regardless of financial or external rewards, has been shown to be key to the performance of public servants. Yet, limited attention has been paid to this kind of motivation in health care settings in low- and middle-income countries. Little is known about PSM and its contextual specificity in the Moroccan health system. We set out to qualitatively explore the meaning of PSM and its expression among health workers in four public hospitals.

**Methods:**

We adopted a multiple embedded case study design to explore PSM in two well-performing and two poor-performing hospitals. We carried out 68 individual interviews, eight focus group discussions and 11 group discussions with different cadres (doctors, administrators and nurses). We carried out thematic analysis using NVivo 10.

**Results:**

Our analysis shows that public service motivation is a notion that seems natural to the health workers we interviewed. Daily interactions with patients catalysed health providers’ affective motives (compassion and self- sacrifice), a central element of PSM. It also provided them with job satisfaction aligned with their intrinsic motivation. Managers and administrative personnel express other PSM components: attraction to public policy making and commitment to public values. A striking result is that health workers expressed strong religious beliefs about expected rewards from God when properly serving patients.

**Conclusion:**

This study highlights the presence of PSM as a driver of motivation among health workers in four Moroccon hospitals, and the prominence of intrinsic motivation and compassion in the motivation of frontline health workers. Religious beliefs were found to shape the expression of PSM in Morocco.

## Introduction

The motivation of health workers is a key concern of policy makers, researchers and practitioners [1, 2], as it is widely regarded as a key determinant of health worker’s performance. Most research on motivation carried out in the field of human resources for health focuses on extrinsic motivation,, performance-based financing and contracting out [3–5]. These latter strategies, inspired by New Public Management principles, have been questioned [6–8] and more specifically in terms of goal-displacement, the risk of crowding out intrinsic motivation and inducing mistrust [9–11]. In response, a number of public management scholars have been developing the notion of Public Service Motivation (PSM). PSM is defined in public management as “*the beliefs, values and attitudes that go beyond self-interest and organizational interest, that concern the interest of a larger political entity, and that motivate individuals to act accordingly whenever appropriate”* [12].

At the core of PSM are motives and actions that are intended to serve the public interest, to serve others and to improve the well-being of society [13]. According to Perry and Wise (1990), three kinds of motives are associated with public service [14]. *Rational* motives refer to the aspiration of workers to participate in good public decision-making, because they consider this as a social duty or because it reinforces their image of self-importance and self-esteem. *Norm-based* motives refer to the desire to altruistically serve the public interest or to serve the nation in order to contribute to social equity [14, 15]. A*ffective* motives involve identification with the public service, affective bonding with service users and compassion, contributing to self-sacrifice [14–16].

The interest in PSM is due to a number of positive effects that have been attributed to it. Research exploring these effects was mainly carried out among public servants in governmental agencies in the USA [17–19], Belgium [20], Switzerland [21], the Netherlands [22], Danemark [23], Malta [24], South Korea [25] and China [26]. The studies indicated significant statistical relationships between PSM on one hand, and individual performance, job satisfaction and reduced turnover on the other. These findings were supported by meta-analysis [27–30] and research designs using quasi-experimental designs [31, 32].

The research on public service motivation started with substantial exploratory research about 20 years ago [14, 18, 33–35]. Later, frameworks were developed that linked policy and management [12] or leadership [36] to PSM. The latter is now considered to be a complex psychological state that is influenced by leadership, organisational and contextual factors [24, 33, 37–40]. Indeed, research has shown that transformational leadership may contribute to PSM [41], but that this is contingent on organisational and contextual characteristics [42–45]. Organisational factors include organisational culture [37, 46] and job characteristics that favour increased contact with public service beneficiaries [31, 32]. Contextual factors include the broader societal culture [47, 48], religion, family education and professionalism [39, 40].

The current state of the art points to the importance of developing robust research methodologies to explore the underlying mechanisms through which leadership, context and organisational attributes may influence PSM [18, 29, 33, 36, 49, 50]. No research, to our knowledge, has explored the concept of PSM in Morocco, nor the contextual factors and mechanisms underlying the emergence of PSM in healthcare settings in Morocco. Yet, cultural differences may explain differences in meaning, antecedents and consequences across countries [46, 51, 52].

The study we present here aimed at exploring the concept of public service motivation in Morocco and how it is expressed by health professionals and managers/administrators working in public hospitals. More specifically, we set out to identify how the notion of public service motivation is being defined by health workers, to explore the differences in the definition by cadre and to explore the factors that may influence PSM.

## Methods

This study is part of a larger research project that adopted the realist evaluation approach [53] to examine the links between leadership, staff motivation and performance. This study is exploratory in nature [54] in a sense that it explores the motivation of health workers using insights from PSM theory in the context of Morocco.

### Setting

Morocco is a lower middle-income country with a population of 35.6 millions [55]. Islam is the religion of most Moroccans and this has an impact on daily life and work place practices [56–58]. The Moroccan society is multi-cultural, collectivistic and strongly attached to family relationships and filial piety [56, 57].

In health, there has been a significant progress in many health indicators (e.g. 35% reduction of the maternal mortality rate between 2010 and 2016), the extension of the coverage to the poor and vulnerable populations, the decentralisation and the introduction of public private partnerships [59, 60]. However, the Moroccan health system remains weak, ranked 134th out 195 countries in terms of health access and quality of care [61] with an inequitable access to care and a poorly regulated private sector [62, 63].

The Moroccan health system is constrained by an acute health workforce shortage, for instance having 0.7 doctors per 1.000 inhabitants and 0.92 nurses and midwives per 1.000 inhabitants) [64, 65]. Studies indicated a lack of staff commitment, poor motivation, low job satisfaction and poor working conditions [58, 66] that have hampered the implementation of many health system reforms, for instance quality assurance programmes [67, 68], fee exemption policies [69]. Other studies found that these health workforce issues affected the quality of patient-provider interactions [70, 71].

### Study design

In this study, we adopted the case study design. The case here is PSM as experienced by health personnel in hospitals. The multiple embedded case study design is appropriate to the exploration of complex phenomena, such as PSM in real world settings and allows for comparison between sites [72]. We purposefully selected two high-performing and two low-performing hospitals. We used the results of the national quality assurance programme called *concours qualité* to select hospitals as our study sites. This programme assessed hospital performance in eight dimensions: (1) accessibility/availability/continuity; (2) patient security and responsiveness; (3) satisfaction; (4) ethics; (5) quality assurance; (6) resource utilisation; (7) technical competencies and (8) leadership. The overall performance score index is measured by the ratio between the actual score assessed by the external audit and the maximum obtainable score. We refer to [67, 68] for an overview of the programme. Using data from the quality assurance report 2011 [73] and 2016 [74], we identified hospitals with a significant increase or decrease of performance between 2011 and 2016.

### Conceptual framework

In the field of public management, the definition of PSM has evolved since 1990, broadening from ‘individual predisposition’ to a more detailed description (Table 1).

**Table 1:** Definitions of PSM

(Perry 1990) [75]	“*An individual’s predisposition to respond to motives grounded primarily or uniquely in public institutions and organizations*.”
(Rainey and Steinbauer 1999) [76]	“*General altruistic motivation to serve the interest of the community, people, state, a nation, human kind*.”
(Brewer and Selden, 2000) [77]	“*The motivational force that induces individuals to perform meaningful public, community, and social service*.”
(Vandenabeele, 2007) [12]	“*The beliefs, values and attitudes that go beyond self-interest and organizational interest, that concern the interest of a larger political entity, and that motivate individuals to act accordingly whenever appropriate*.”

In the last two decades, most PSM research focused on developing measurement scales. These studies were carried out in the USA, Europe, Asia and South America [78]. Wright noted a high degree of variability in operational definitions and a diversity of PSM scales [79]. To overcome this diversity, Kim and colleagues [80] refined the multidimensional scale developed by Perry and Wise [35] and validated it across 12 industrialised countries [81–83]. It comprises:

- Rational motives
  - attraction to public service, which means a “disposition to serve the public, to work for the common good, and to participate in public policy processes” [15].
- Norm -motives
  - commitment to public values, understood as “a personal disposition to pursue public values” [15].
- Affective motives
  - compassion, defined as “an affective bonding with identified objects, such as other members of a social category or of a political system” [15].
  - self-sacrifice, meaning “the willingness of public servants to forego financial rewards for the intangible rewards they receive from serving the public” [35].

In this study, we consider individual motivation as mix of motives including a continuum that ranges from extrinsic motivation to more autonomous forms of motivation (intrinsic motivation and PSM). We mean by intrinsic motivation ‘*the doing of an activity for its inherent satisfaction rather than for some separable consequences’* [84]. We adopt Ryan and Deci’s definition of extrinsic motivation: “*a construct that pertains whenever an activity is done in order to attain some separable outcome*” (e.g. tangible and verbal rewards) [84]. We adopted the definition of Vandenabeele of PSM. As explained below, we used the 4 components of PSM as defined by Kim et al. [83] and the concepts of intrinsic and extrinsic motivation as defined by Ryan and Deci [84] to analyse the data.

### Data collection and analysis

We started with interviews and then conducted group discussions. A document review was carried out all along the study. All data were collected during the period January–June 2018 (see Tables 2 and 3).

**Table 2:** Data collection

	EJMH	NHMH	RKMH	SMBA
In-depth Interviews	17	18	16	17
Focus group discussions	3	1	1	3
Group discussions	2	2	2	5

**Table 3:** Respondents characteristics

	Individual Interviews				Group discussion				Focus Group Discussion				Total
	EJMH	NHMH	RKMH	SMBA	EJMH	NHMH	RKMH	SMBA	EJMH	NHMH	RKMH	SMBA	
a. Professionnal Profile													
Doctors	5	10	3	8	3	3	0	2	6	0	0	4	44
Pharmacist	1	1	1	0	2	0	0	1	0	0	0	0	6
Nurse	8	6	7	8	1	0	3	5	6	8	5	7	64
Administrator	3	1	5	1	0	3	2	3	8	0	0	6	32
Total	17	18	16	17	6	6	5	11	20	8	5	17	146
b. Managerial Position													
Senior	4	4	2	4	0	0	0	0	0	0	0	0	14
Intermediate	7	2	1	1	0	1	0	0	0	0	0	0	12
Operational	1	4	5	2	0	0	0	0	0	1	0	0	13
Total	12	10	8	7	0	1	0	0	0	1	0	0	39
c. Age category													
20–30	0	2	1	1	0	0	2	1	3	4	2	1	17
31–40	7	4	3	3	2	3	1	6	2	4	2	8	45
41–50	2	8	7	8	3	1	2	1	5	0	0	2	39
51–63	8	4	5	5	1	2	0	3	10	0	1	6	45
Total	17	18	16	17	6	6	5	11	20	8	5	17	146
d. Gender													
F	6	10	5	4	4	4	3	7	15	6	3	13	80
M	11	8	11	13	2	2	2	4	5	2	2	4	66
	17	18	16	17	6	6	5	11	20	8	5	17	146

#### Interviews

We started the data collection by interviewing hospital staff. In total, we carried out 68 in-depth interviews (IDI) with senior-, middle-, operational-level managers and health professionals [85, 86]. We explored the views of respondents on public service motivation, its definition and its expression, as well as the factors that may influence PSM. We used adapted open-ended interview guides for each category of respondents (Additional file 1). These were tested in a pilot study with professionals. We carried out the interviews until saturation was attained.

#### (Focus) group discussions

In a second stage, we carried out eight focus group discussions and 11 group discussions with different cadres (administrators, nurses and doctors) to further explore the key constructs mentioned by interviewees in relation to motivation. This allowed us to deepen the analysis across different categories of health workers (Table 3). The FGD and group discussions were conducted by the first author, who used a facilitator guide (Additional file 2). We conducted the FGD following standardised procedures described by Krueger and Morgan and used probes, asked follow up questions, summarised key themes and sought verification from participants [87, 88].

We used qualitative purposive sampling [86] in order to identify respondents for the in-depth interviews and the focus group discussions. Interviews were carried out in Moroccan dialect. All interviews, FGDs and group discussions were audio-recorded with the exception of two interviews. In these specific cases, we took notes and transcribed the unrecorded interviews using memory recall [85]. All transcripts were checked for accuracy by two co-authors (ZB and BM).

Table 2 presents the breakdown per hospital. We used codes to identify respondents anonymously, referring to the hospital EJMH, NHMH, RKMH, SMBA.

At the end of each contact with research participants, we wrote a brief contact summary that included major themes and ideas arising from the interaction following guidance provided by Miles and Huberman, and Krueger [87, 89]. Table 3 represents the summary of data collection tools and respondents’ profiles (Additional files 3, 4, 5 and 6 provide detailed descriptions of the respondent characteristics).

#### Document review

In order to identify key elements in the broader health policy context and to describe the general context, we collected documents all along the study. Key informants at the four hospitals and at the Ministry of Health contributed to identifiying relevant documents. We focused on human resources availability and skill mix data, strategic plans, audit documents and quality assurance reports.

### Analysis

We structured the qualitative data analysis along the analytic phases of compiling data, interpreting data, discussion and drawing conclusions [90]. During the initial coding cycle, we coded all data sources (transcripts, contact summaries and field notes) using different coding techniques (concept, hypothesis and “in vivo” coding) [91] and used NVivo 10 v11. 4.3 software to manage the data [92]. During the second coding cycle or pattern coding, [89], we used the four PSM components (attraction to public service, commitment to public values, compassion, self-sacrifice) described by Kim and colleagues [83] and the intrinsic/extrinsic types of motivation defined by Ryan and Deci [84]. As we will present below, we also identified other categories of motives that emerged from the inductive analysis of data (religious based motives). The coding was discussed during research team meetings. These meetings were conducted at different moments during the analysis, focusing on the initial coding, the second coding cycle, the in-case analysis and the cross-case analysis.

### Ethical considerations

The research protocol was approved by the Moroccan Institutional Review Board in Rabat (n°90/16) and the Institutional Review Board of the Institute of Tropical Medicine, Antwerp (N° 1204/17). All interviewees were informed before the start of data collection about the study objectives, the topics, the type of questions and their right to refuse being interviewed or interrupt the interview at any time. The same information was included in an information sheet that was given to candidate interviewees and reiterated when the written consent form was discussed before the start of the interview. The informed consent forms were signed by the participants and co-signed by the researcher. A copy of the signed consent form was given to research participants.

## Results

In this section, we present how health workers belonging to different cadres define ‘public service motivation’ and identify which factors may influence the level of PSM. We start with a summary of the intrinsic and extrinsic motives of the respondents.

### What motivates health workers?

We found that the respondents were motivated by a mix of intrinsic and extrinsic motives.

#### Intrinsic motives

Both nurses and doctors expressed the importance of their intrinsic motivation, which is fuelled by the satisfaction derived from applying their professional skills and competencies.

“I love my job. I chose deliberately to work at the emergency unit. I love working at the emergency unit. I am totally engaged. Handling serious medical emergencies is a motivation in itself”. EJMH 38, Doctor.

“I am frustrated because my salary does not compensate my efforts. However, nursing is a noble profession that has nothing to do with financial incentives.” NHMH 30, Nurse.

Performing non-clinical tasks such as participating in quality circles and community volunteering aligned with intrinsic motivation. This enhanced their feeling of self-efficacy.

“As for me, this [participating in the quality contest] was a great pleasure. I enjoyed that. This was not for the sake of doing good for others, but it was mainly for my own satisfaction… My objective was to accomplish this managerial task and to prove to myself that I can do this. This is why I was striving to make that effort.” NHMH 10, Doctor.

“I participated in several ‘medical caravans’. This was for me just pleasure. I just enjoyed it. It was not about the feeling to do good for others. I gained patient recognition and above all I felt self-efficacious toward patients”. NHMH 31, Doctor

#### Extrinsic motives

More extrinsic motivation-related drivers were reported as well, including the importance of recognition by leaders and patients.

“We need that leaders recognise our performance: 6.000 deliveries a year! We need that they congratulate us”. SMBA 06, Midwife.

“We are satisfied when our effort is acknowledged by others (patients).” NHMH 13, Doctor

“When people came to thank me, because they get well because of me, I feel that I am the happiest man in the world. Because, I hate to walk around in the city and that people bad mouth me: “This obnoxious doctor did not treat me well”. I cannot tolerate this.” SMBA 18, Doctor.

Esteem from supervisors was highly valued and led some health workers to feel guilty when they do not meet their supervisors expectations.

“I feel ashamed, if I did not do the work my superior ask me to do”. SMBA 05, Nurse / senior manager.

Other sources of extrincic motivation mentioned by our respondents include job security, flexibility of working schedules and work-family life balance.

“Here in the public sector, there is a certain liberty, work is fluid. This is why I choose the public sector. I avoided the private sector where I could earn twice or thrice my salary, but I dropped the economic reasons, and chose to get the minimum wages offered in the public sector, because I chose to be free, because there is less hierarchy than in the private sector or in the university teaching hospital, where the doctor in chief could sanction me severely and even stop my salary.” NHMH 2, Nurse.

“Why am I staying at the public sector? I can earn three times my salary in the private sector. Well, I have a six years old daughter, I need to enjoy her company.” RKMH 3, Doctor.

### How is PSM defined?

It emerged from our analysis that public service motivation is a notion that seems natural to the health workers we interviewed. When talking about PSM, respondents mentioned several components: affective motives (compassion and self-sacrifice), normative motives (commitment to public values) and rational motives (attraction to public service). In addition, they reported religion-based motives they labelled as ‘seeking divine rewards’.

#### Affective motives

In terms of affective motives, our respondents talked mainly about compassion and self-sacrifice. To a striking degree, nurses and physicians expressed compassion with patients’ conditions as a major motivational factor. This emotional response was also expressed by administrative personnel with frequent contacts with patients (e.g. cashiers).

“Patients are important for me because I got sick. So, I sense what the patients are feeling. My family members, my daughter and my grandmother got sick. So, I feel the pain patients are suffering from. I can feel their suffering. ” SMBA 35, Nurse.

Staff were expressing compassion with the vulnerable and underprivileged members of the population. They were placing themselves in their situation and showing empathy toward these patients and their families. This even led some of them to help patients pay user fees.

“One day, a citizen came to pay for laboratory tests for his daughter who had fever and he could not afford to pay the fees. I added the missing amount from my own money. Not every day, but often, I bring small change to help citizens who do not have the full amount. My wallet is always opened.” NHMH 29, Revenue officer.

Respondents explained that serving the underprivileged and caring for the poor is one of the reasons that kept them working in the public sector.

“Here, I work a lot with vulnerable citizens. It is a reward in itself to serve poor patients. It is my source of motivation”. RKMH 3, Doctor

The notion of self-sacrifice was mentioned but not often. Some respondents expressed the importance of serving the patient compared with the financial rewards. Some health workers may forgo their own health needs in order to serve patients in what we call an “escalation of commitment”.

“Sometimes, I think I sacrifice a lot in order to serve others. We cannot justify this. It is not reasonable. It is 16h30 and I just ate! I do not eat. I lost weight, I got tuberculosis, I had cervical pain, arthralgia, backpain, a sciatica. I had thoracic pain because I suffered from a pleural effusion. Is it logical to suffer in order to treat patients?” EJMH 38, Doctor Emergency Unit.

“The cardiologist is highly concerned about patients. She is worried about them. Even if her shift ends at 4H30 pm, she keeps coming back to the hospital to check on them, even during weekends. She is continuously in contact with us by phone to check on her patients, their test results, their condition. She is omnipresent. If a patient is in a bad condition, she returns to the hospital. She does not have to, but she keeps coming back even during weekends. She is so consciencious and keeps checking on her patients. This is why she got sick!” EJMH 24 nurse.

#### Norm-based motivation

In terms of norm-based motivation, our analysis shows that many respondents emphasised their desire to serve the public interest and their high sense of civic duty. They referred to these motives as feelings of citizenship, the desire to serve the nation, the citizens and the general interest, and the need to be rightful and equitable. This was expressed by all categories of staff (doctors, nurses and administrative staff).

“A love for our country, love of our territory, love of the land of our ancestors, love of neighbourhoods, we love to leave a suitable environment for our siblings.” EJMH 8, Administrator.

“We are Moroccans serving Moroccans. I do not feel proud if I could not serve adequately our citizens. We are an integral part of this institution. The reputation of this institution is our reputation. We are a small cell within a large cell that is the Ministry of Health. We work with devotion”. NHMH 16, Doctor.

Some of the health workers who expressed a commitment to public values described how that led to prosocial behaviours, such community volunteering.

“We care for the public service. We organized a community action in the former hospital location on a voluntary basis, without any instruction from the hospital administration. We noticed that the hospital garden was deteriorating. So, with other friends and union representatives, we refurbished the garden.” EJMH 23, Nurse.

“I worked for non governemental organisations, doing medical caravans with doctors in rural areas in Walidia. I started working for NGOs not affiliated to any political party in 1979-1980. I believe that working for NGOs educates young people. We grew up doing medical caravans, serving the population, we worked with doctors till 8 or 9 pm.” EJMH 41, Nurse.

#### Rational motives

Finally, we examined in how far respondents mention rational motives for working in the public service. We found that nurses who occupy managerial positions emphasised the importance of participating in decision-making and the social importance of their function. They feel proud when they are consulted and involved in decision-making. For some, this contributes to their motivation, as it allows them to more effectively serve the public.

“As the chief of this department, the most important thing for me is when I am involved in decision-making. I do not only report on problems. As a leader, I suggest solutions that get always approved by the hierarchy.” EJMH, 12 Nurse intermediate manager.

“This title [Chief Nursing Officer] allows me to commit to my job. As a chief nursing officer, I have an authority on all nurses in the hospital. Sincerely, this title motivates me because I am solicited by staff to provide them with the necessary support. This is true for both doctors and nurses. I am fully satisfied when my opinion is listened to and taken into consideration. This makes me more motivated to fill this position that allows me to serve people.” EJMH1, Senior manager, Nurse.

PSM was expressed similary by respondents from both poor- and high-performing hospitals. However, in poor performing hospitals, staff said they were suffering from psychological distress and feelings of guilt because of their inability to perform their job adequately and to ease their patients suffering.

“We lose patients stupidly because of a lack of material. There is no material to work with. You see patients die in front of you and you do not have necessary tools to save them. These conditions are beyond our control.” SMBA 43, Doctor

This influenced negatively their well-being.

“When you do not have necessary material to work with, you are in trouble! It is not only a constraint, but a source of suffering. Instead of relieving patients’ distress, it is us who get stressed.” SMBA 45, Doctor

### What contributes to public service motivation?

The respondents in both high- and poor-performing hospitals identified a number of factors that contribute to high PSM: family education, military service, volunteering, professionalism and religion.

#### Family education

Health workers with a high level of PSM explained how family education and childhood experiences contributed to their high sense of civic duty and a high orientation to civic participation. Parent modelling and education led them to serve others and to act for the common good from a non-self-interested perspective.

“I do not take bribes. I have an ideal about the role of doctors in society. Their role is not limited to being a care provider at the hospital. They should get out to the community, sensitize the population during health education sessions. I feel satisfied when we organize a round table with practitioners and local representatives, when we organize medical caravans, when we circumcise children for the sake of God. I learned these principles through my parents’ education. When we were young, we were educated to help people, to help others, neighbours, friends, siblings and family members. I cannot explain these things, I do not know if it is genetics, but we learn that we do not live alone but in a society. We depend on each other, we belong to a society, we live with neighbours and people. Everybody is leaning on others, like dominos.” SMBA 40, Surgeon

#### Military service

Some health workers explained that their commitment to public values is reinforced by their former experience in the mandatory military service. These experiences provided them with high sense of civic duty and citizenship, which subsequently contributed to their feelings of public service motivation.

“Every health worker, specifically males, should do military service, as I did. At that time, I went to Al Farssia, a remote area in the Moroccan territory in the middle of nowhere. I used to stay there between two to three months and then would go back to town for a short period of time. Every time I went back to that region, I recognized the true meaning of life and the true value of things, when I compared urban life to these remote areas.” NHMH 32, Doctor/Senior Manager.

“I was asked to join the military service. … I believe that in my country, we are more effective if we serve the poor, if we work for the interest of the most vulnerable citizens. The experience I had in the medical service in the military affected me a lot. It has shown me that Morocco needs more faithful and serious health workers.” SMBA 18, Doctor

#### Volunteering

The experience with volunteering in remote areas during medical outreach caravans shaped the feelins of PSM of some respondents. They identified this as a catalyser of self-sacrifice and civic participation.

“From 1970 to 1980, I was part of an NGO doing medical caravans with physicians in rural areas. Volunteering educates people. When you grow up volunteering, you will be loving to serve the population. I worked for 8 to 9 hours a day without being paid. I did it for the sake of God without waiting for external rewards. EJMH 41, Nurse.

#### Professionalism

Some respondents value the comfort of the patients and expressed a strong sense of ethical responsibility towards them. They related this to their professional ethics and believe this attitude is embedded in their professional identity. They asserted that their behaviour is less dependent on the supervision of their superiors than it is relying on their professional conscientiousness.

“I love my job. I cannot neglect my job. This is how I was educated and taught in the first place. I cannot let down a patient, even though I know that this (quality of care) does not depend only on me. I cannot. I try to help patients even if they are from other departments. I call the surgeon to deal with a patient with a suspicion of appendicitis, even if it is the job of the physician at the emergency department.” SMBA 24, Nurse,

Their commitment to serve the patients is integrated within their professional identity but also with their religion.

“ ‘Citizens’ means for me ‘professional conscientiousness’ [in Arabic, *damir*]. I have to serve them conscientiously. It is important part of my personality. I do not like to fail to properly perform my duty. My money will be then *halal* [Compliant with what is permitted in Islam]. NHMH 13, Nurse

#### Religious beliefs

Our analysis shows that quite some interviewees expressed strong religious beliefs about expected rewards from “God” when properly serving patients. They explained their altruistic behaviour by their expectance of divine rewards (called *ajre* in Arabic) when serving patients. This was expressed by nurses, administrators and doctors alike.

“He [the patient] praises God for you, he says nice prayers! “May Allah [God] be pleased with you. May Allah be merciful to you. May you be covered by the grace of God”. At such moments, I feel reassured and relieved during my night shift. Then I go home fully satisfied.” SMBA 35, Nurse.

“When serving people, you earn *ajre* (divine rewards) from God, which is far better than money.” EJMH 18, Administrative staff.

‘This is between us and God, Glory be to Allah. It is between me and God who created me. We do good deeds when serving patients because we need *ajre* [divine rewards]. We do not know what might occur to us in the future. We seek *ajre* from God. He is the only one who will reward us for our effort”. SMBA 7, Nurse.

Our analysis indicates that religious beliefs may underlie public service motivation to the extent that these beliefs contribute to an altruistic and compassionate attitude towards patients as well as to commitment to public values.

“There is a religious element that plays a role in my motivation. We have to make our money *halal* [meaning in compliance with Islamic rules]. This means I have to work, to serve people. We are equal to them. We are all Muslims. They [patients] are our fellow countrymen, our fellow citizens. We are obliged to give them back what we owe to our country.” NHMH 11, Surgeon, Intermediate-level manager.

Our data show that these religious beliefs are closely aligned to the professional sense of responsibility and duty, to the attraction to public service and to compassion.

“I am pleased when I treat patients decently and they pray God for you. I am pleased when I see that patients are happy and in a good state.” SMBA 35, Nurse

“Have mercy on those on earth, the one in Heaven will have mercy on you ... This is our real profession, to love others. I am a human, I can also become sick. We are all patients”. SMBA 17, Nurse

We found that for many health workers we interviewed, PSM and religious beliefs and values are intertwined. They express their altruistic and compassionate attitude towards patients as a spiritual personal obligation. When serving patients ethically and altruistically, they expect to receive intangible and divine rewards (in arabic *hassanat*’ or *ajre).* They also frame this as a commitment to public values such as equity and patriotism.

“In Arabic terms, we do this because we need to make our salary “*Halal*.” [*Halal* means compliant with Islamic rules]. “Thank God.” We said this because it is very important! We must make sure that our salary is *halal* before God and our own conscience. There is nobody watching over us, whether we came at 8 am in the morning or not”. EJMH 9 doctor

When discussing PSM, health workers also referred to the ‘*Hadith*’, the sayings of the Prophet Muhammed, when they justified why they are committed to help patients and the underprivileged.

“[In the Prophet Muhammed sayings and teachings], the prophet said “Who served other muslims, is rewarded like someone who stayed a month praying in this mosque.” This explains why I came early at 8h30 am. Sometimes, when I come late to work, I stay late in compensation, hoping that this way, God will help my siblings.” NHMH 11, surgeon

“We are muslims. Then, satisfying the need of others is essential [referring to ‘*Hadith*’ ]. I feel satisfied and I enjoy that. We have an ideology that dominates our behaviour as muslims. We are not compensated directly but we get rewards later in other circumstances. Sometimes, I might serve and help this old lady we have just seen. By doing so, I might be helped in the future when I will experience the same situation.” NHMH 7, Doctor.

## Discussion

In this study, we explored how health workers from four Moroccan hospitals describe public service motivation. We found that most respondents expressed some form of public service motivation, both in the high- and the poor-performing hospitals. Our respondents referred to the main elements identified by Kim et al.: compassion, self-sacrifice, commitment to public values, and attraction to public service.

In our four sites, we could clearly distinguish between administrators on one hand and health professionals (nurses and doctors) on the other. We found, in line with other study findings [32, 93, 94], that health professionals identified compassion and self-sacrifice as major components of PSM, while administrative staff (except those in direct contact with patients) and managers tended to indicate commitment to public values, besides feeling attracted to policy-making. Our analysis suggests, similarly to other PSM studies [32, 93–96], that the nature of professional work and the daily interaction with patients catalyses affective motives among healthcare providers. This is conditioned by the ability of health workers to help and ease the suffering of patients. In our study, similarly with other empirical findings [16, 97, 98], we found that health workers experiencing unnecessary deaths or harm to patients expressed high levels of psychological distress because of these events but also because of the conflict with their public service motivation.

Our study showed that managers, and nurses in management positions in particular, similar to previous studies [46, 99], valued the importance of social status and respect from other staff. This in line with the dominant organisational culture in the Moroccan public sector, which emphasises the importance of social status [100–102].

Although PSM scholars usually do not consider that intrinsic motivation is an essential part of PSM [103], our study showed that health professionals are motivated by both PSM and intrinsic motivation and that both are sometimes difficult to disentangle. We found that doctors and nurses were highly intrinsically motivated by the task of caring and saving patients’ lives. They enjoyed the perceived self-efficacy and the esteem from patients and their relatives. We tend to agree with Grant and Berry [104, 105] that intrinsic motivation is essential in maintaining the persistence of PSM in medical and nursing professions: the day-to-day interactions of these providers with patients shape and reinforce their helping role identity and this contributes to PSM [32, 106, 107]. Our analysis showed that PSM and intrinsic motivation are related to personal volition: the locus of causality is internal. In that sense we agree with Perry (1990) that health workers have a mix of motives: PSM, intrinsic motivation and extrinsic motives (supporting their families, job security and stability, work family balance). The relative importance at any given time in one’s career depends on contextual conditions and personal factors. This is line with findings from other studies in North African countries [101, 108, 109].

We also found that a number of respondents framed their public service motivation in religion-based motives, such as divine rewards (and Islam in general) and with roots in family education. This is similar to findings of studies in Morocco [56, 110–112], Egypt [102], and Tunisia [101]. In as far as religion and family education are social institutions that shape the identity of health workers before they enter the public health sector, these can be considered as contextual elements. Future research could focus on the underlying mechanisms by which religion and education influence the formation of health workers’ identity and how that translates (or not) in PSM. Indications about this relationship is found in studies carried out in industrialised countries [38–40, 113–117].

As mentioned in the introduction, this study on PSM is part of a larger project looking into the relationship between leadership, motivation and performance. Future research needs to examine the link between PSM and individual and hospital performance. We think managers of public hospitals should be aware of the differences in PSM among health workers and adapt their leadership practices accordingly. In the Moroccan context, they may need to emphasise spiritual and public values when communicating about organisational mission and objectives, and reinforce the interaction of their early-carrier health personnel with the underprivileged parts of the population during, for instance, medical outreach and other community-level initiatives. Finally, they also may ensure that adequate resources are provided for frontline healthworkers to allow them to experience valued outcomes such as saving patients’ lifes.

This study has a number of limitations. First, we did not measure actual levels of PSM, largely because of the time constraints which precluded validation of existing scales in Morocco. This study was thus necessarily exploratory in nature. Further studies should indeed validate, and adapt if necessary, the scales of for instance Kim et al. [83] in order to substantiate our results. Second, it would have been interesting to interview health workers who left the public service to explore their views on PSM. We also acknowledge that in hospital NHMH and RKMH, four planned FGDs were in practice carried out as group discussions because the necessary number of participants (6 to 8) was not reached.

## Conclusion

While the notion of PSM is not part of the management discourse in Morocco, we found that PSM seems to be a ‘natural’ concept to health workers in Moroccan public hospitals. We found that hospital staff are motivated by different drivers of PSM and that religious-based beliefs infuse the notion of public service. Hospital managers should pay more attention to the nature of staff motivation (PSM, intrinsic and extrinsic) and adapt their leadership practices accordingly.

## Supplementary information

**Additional file 1:** Open ended interview.

**Additional file 2:** Focus Group Discussion Guide (senior managers).

**Additional file 3:** Sociodemographic characteristics, case study 1 (NHMH).

**Additional file 4:** Sociodemographic characteristics, case study 2 (EJMH).

**Additional file 5:** Sociodemographic characteristics, case study 3 (RKMH).

**Additional file 6:** Sociodemographic characteristics, case study 4 (SMBA).

## Data Availability

Not Applicable.

## Abbreviations

LMIC: Low and Middle Income Countries
OECD: Organisation for Economic Co-operation and Development
PSM: Public Service Motivation
