# Supplementary material for: Does public service motivation matter in Moroccan public hospitals? A multiple embedded case study
Source: Int J Equity Health. 2019 Oct 22;18:160. doi: 10.1186/s12939-019-1053-8 (PMC6805632; doi:10.1186/s12939-019-1053-8)
Supplement: Supplementary file 1 — Additional file 1: Open ended interview. [file 12939_2019_1053_MOESM1_ESM.docx]

Additional file 1: Open ended interview

This interview topic guide gives an indication of the main questions that will be asked in the interviews of health service managers and providers. Core questions were adapted to meet the specificity of each category (senior managers (Core questions 1 to 4), intermediate managers (Theme 1 to 5); health professional (Theme 2 to 5).

| Components | Objectives/Remarks / Questions |
| --- | --- |
| Introduction | Researcher presentation (Name, qualification, institution) |
|  | Interview objectives |
|  | Explain the procedure (Time, Clarification questions, information about voluntary participation and the autonomy to respond or not to sensitive question and information about consent forms) |
|  | Explain confidentiality and data anonymisation procedures |
|  | Ask permission to record the interview (Audio record and notes) |
|  | Obtain informed consent |
| Adjust the recording device | Make sure that equipment is functioning, and the room is not noisy |
| General part | To get overall idea about the interviewee and make him/ her comfortable |
|  | Q: How old are you? |
|  | Q: Could you describe your actual job position? Your tasks? |
|  | Q: How long have you been working in your actual position? |
|  | Q: How long have you been working in this hospital? |
|  | Q: Where have you worked before? In which function? |
| Introduction to specific questions | Transition to core questions |
| 1) Leadership Practices | Q: Could you describe you task? |
|  | Q: Could you describe your role as a manager? P |
|  | Q: What is your vision about leadership? What do a good leader means to you? |
|  | Q: Would you give me some examples of your practice of leadership? |
|  | Q: What challenges are you confronted with in your leadership practice? |
|  | Q: In your opinion, how could you describe your influence on staff behaviours? |
| 2)Hospital Performance | Q: In your opinion, what explain the good/bad performance of your hospital in "Concours Qualité”? |
|  | Q: Is it related to leadership? Does leadership matters? |
| 3) Individual Performance | Q: In your opinion, what are the major reasons why a health professional is performant in health care provision? |
|  | Q: According to you, what are the facilitators to individual performance? |
|  | Q: In your opinion, what are the barriers to maintain a good individual performance for health professionals ?. |
|  | Q: Is there a difference in the motivation between different cadres of health professionals or not? |
|  | Q: How could you play a role in the motivation of your staff/ colleagues? |
| 4) Public Service Motivation | Q: Could you explain what motivates you to work in this hospital? (Motivation intrinsic/extrinsic) |
|  | Q: how do you feel working in this hospital? |
|  | Q: What attaches you to this hospital, if any? Q: how do you describe this attachment? |
|  | Q: serving citizens, what does it means for you? |
|  | Q: Did you think about quitting the public service? If yes, why? If no, why? |
|  | Q: Do you feel that you are doing tasks that go beyond your responsibilities, or not? |
|  | Q: how could you describe your engagement about the organisational mission and vision? |
|  | Q: Do you feel that you have the necessary information, tools and support to carry on your task, or not? |
|  | Q: Do you engage in supplementary efforts without contingent financial rewards? Could you give me some examples? |
| 5) Leadership in your organisation | Q: Could you describe leadership practices in your organisations? |
|  | Q: Do you feel that you are supported by your superior? By management teams? |
|  | Q: Could you provide some examples of leadership practices of your superior? |
|  | Q: How do you describe the relationship between your interaction with your leader and your motivation? |
| Summary and debriefing | During this interview you gave me useful information that are relevant to this study. |
|  | Q: Is there something that you see as important regarding our topic we did not mention? If Yes, we could discuss it. We do have time. |
|  | Q: Do you have questions for me? |
