# Supplementary material for: Does public service motivation matter in Moroccan public hospitals? A multiple embedded case study
Source: Int J Equity Health. 2019 Oct 22;18:160. doi: 10.1186/s12939-019-1053-8 (PMC6805632; doi:10.1186/s12939-019-1053-8)
Supplement: Supplementary file 2 — Additional file 2: Focus Group Discussion Guide (senior managers). [file 12939_2019_1053_MOESM2_ESM.docx]

Additional file 2: Focus Group Discussion Guide (senior managers)

| Themes | Questions | Prompts, clarifications, vignettes |
| --- | --- | --- |
| Motivation | Q 1: What motivates at work at this hospital? |  |
|  | Q 2: How do you feel at work at the hospital? |  |
| Public Service Motivation | **Q2: Why did you choose to the work at the public sector?** You told me about your (de) motivation in the public sector? Could you explain your (de) motivation? |  |
|  | Q3: Serving citizen, what does it mean to you?  Give me examples from your professional experience? |  |
|  | Q 4: Did you think about quitting the public service? If Yes, why? If no, why not? | **Vignette 1** Mr or Dr Rachid work in this hospital for 10 years, he did not leave the public hospital to work in the private sector because he feels satisfied with the help, he is providing to the local underprivileged population What do you think about Dr /Mr Rachid perspective? |
|  | Q 5: Do that you are well paid according to your contribution to this hospital? If Yes, why? If no, why? | **Vignette 2**: Dr/Mr Rachid a has accompanied many patients in medical transfers although he is not well remunerated. he continues to do it when asked. What do you think about his attitude? |
| Leadership | Q6: in your opinion, what does it mean a good leader? | **Vignette 3: A** manager told me that leadership is important in the motivation of staff. Do you agree with that.? |
|  | Q 7: How could you describe the leadership of your supervisors? | Do you agree that leadership play a role in the staff performance? |
|  | Q 8: Does managers' leadership matters for you to be performant at work? |  |
| Interaction | Q 9: How would you describe the relationship between your interaction with the leader and your motivation? |  |
| Leadership- Motivation |  |  |
| Organisational performance | Q: According to you, what explains the good/ bad performance of your hospitals in "Concours Qualité"? | Who was involved? Who took leadership roles? Who was responsible for decision making? |
|  | Q: What makes you perform well/bad under the leadership of Mr/Mme ? |  |
