# Supplementary material for: Does public service motivation matter in Moroccan public hospitals? A multiple embedded case study
Source: Int J Equity Health. 2019 Oct 22;18:160. doi: 10.1186/s12939-019-1053-8 (PMC6805632; doi:10.1186/s12939-019-1053-8)
Supplement: Supplementary file 3 — Additional file 3: Sociodemographic characteristics, case study 1 (NHMH). [file 12939_2019_1053_MOESM3_ESM.docx]

Additional file 3: sociodemographic characteristics case 1

| Code | Age | Managerial function | Professional profile | Genre |
| --- | --- | --- | --- | --- |
| NHMH 1 | 41-50 | Senior Manager | Doctor (General Practictionner) | Female |
| NHMH 2 | 31-40 | Non Manager | Nurse anthesiologist | Male |
| NHMH 3 | 41-50 | Non Manager | Doctor (General Practictionner) | Male |
| NHMH 4 | 20-30 | Non Manager | Nurse | Female |
| NHMH 5 | 31-40 | Intermediate Manager | Pharmacist | Female |
| NHMH 6 | 41-50 | Non Manager | Doctor (Specialist) | Male |
| NHMH 7 | 41-50 | Senior Manager | Doctor (General Practictionner) | Male |
| NHMH 8 | 31-40 | Operational Manager | MidWife | Female |
| NHMH 9 | 51-63 | Operational Manager | Nurse | Female |
| NHMH 10 | 51-63 | Non Manager | Doctor (Specialist) | Male |
| NHMH 11 | 41-50 | Non Manager | Doctor (Specialist) | Male |
| NHMH 12 | 31-40 | Operational Manager | Administrator | Female |
| NHMH 13 | 20-30 | Non Manager | Nurse | Female |
| NHMH 14 | 31-40 | Non Manager | Administrator ( former nurse) | Female |
| NHMH 15 | 31-40 | Non Manager | Administrator ( was a nurse) | Male |
| NHMH 16 | 51-63 | Operational Manager | Doctor (General Practictionner) | Female |
| NHMH 17 | 20-30 | Operational Manager | Nurse | Male |
| NHMH 18 | 20-30 | Non Manager | Nurse | Female |
| NHMH 19 | 31-40 | Non Manager | Laboratory technician | Female |
| NHMH 20 | 31-40 | Non Manager | Nurse anthesiologist | Female |
| NHMH 21 | 31-40 | Non Manager | rAdiology technician | Female |
| NHMH 22 | 31-40 | Non Manager | Nurse | Female |
| NHMH 23 | 20-30 | Non Manager | Nurse (Operating theator) | Male |
| NHMH 24 | 20-30 | Non Manager | Nurse anthesiologist | Female |
| NHMH 25 | 51-63 | Intermediate Manager | Doctor (General Practictionner) | Male |
| NHMH 26 | 41-50 | Non Manager | Doctor (Specialist) | Female |
| NHMH 27 | 31-40 | Non Manager | Doctor (General Practictionner) | Female |
| NHMH 28 | 51-63 | Non Manager | Doctor (Specialist) | Female |
| NHMH 29 | 51-63 | Non Manager | Cashier (Technical staff) | Female |
| NHMH30 | 41-50 | Senior Manager | Nurse | Male |
| NHMH31 | 41-50 | Intermediate Manager | Doctor (General Practictionner) | Female |
| NHMH32 | 41-50 | Senior Manager | Doctor (Specialist) | Male |
