# Supplementary material for: Does public service motivation matter in Moroccan public hospitals? A multiple embedded case study
Source: Int J Equity Health. 2019 Oct 22;18:160. doi: 10.1186/s12939-019-1053-8 (PMC6805632; doi:10.1186/s12939-019-1053-8)
Supplement: Supplementary file 4 — Additional file 4: Sociodemographic characteristics, case study 2 (EJMH). [file 12939_2019_1053_MOESM4_ESM.docx]

Additional file 4

| Code | Age | Managerial function | Professional profile | Genre |
| --- | --- | --- | --- | --- |
| EJMH 1 | 41-50 | Senior Manager | Nurse | Female |
| EJMH 2 | 41-50 | Senior Manager | Doctor (General Practictionner) | Male |
| EJMH 3 | 31-40 | Intermediate Manager | Administrator | Male |
| EJMH 4 | 51-63 | Non Manager | Pharmacy technician | Female |
| EJMH 5 | 31-40 | Non Manager | Pharmacist | Female |
| EJMH 6 | 31-40 | Non Manager | Pharmacist | Female |
| EJMH 7 | 51-63 | Senior Manager | Doctor (General Practictionner) | Male |
| EJMH 8 | 51-63 | Non Manager | Administrator | Male |
| EJMH 9 | 41-50 | Non Manager | Doctor (General Practictionner) | Male |
| EJMH 10 | 41-50 | Non Manager | Doctor (General Practictionner) | Female |
| EJMH 11 | 41-50 | Non Manager | Doctor (Specialist) | Male |
| EJMH 12 | 31-40 | Intermediate Manager | Laboratory technician | Female |
| EJMH 13 | 51-63 | Intermediate Manager | Doctor (Specialist) | Male |
| EJMH 14 | 31-40 | Intermediate Manager | Nurse | Female |
| EJMH 15 | 41-50 | Non Manager | Technician (Technical staff) | Female |
| EJMH 16 | 41-50 | Non Manager | Administrator ( former nurse) | Female |
| EJMH 17 | 41-50 | Non Manager | Administrator (former nurse) | Female |
| EJMH 18 | 51-63 | Non Manager | technician (Technical staff) | Female |
| EJMH 19 | 20-30 | Non Manager | Technician (Technical staff) | Female |
| EJMH 20 | 51-63 | Non Manager | Administrator | Female |
| EJMH 21 | 41-50 | Non Manager | Administrator (former midwife) | Female |
| EJMH 22 | 51-63 | Non Manager | Administrator | Female |
| EJMH 23 | 51-63 | Intermediate Manager | Nurse | Male |
| EJMH 24 | 31-40 | Non Manager | Nurse | Female |
| EJMH 25 | 31-40 | Intermediate Manager | Pharmacist | Male |
| EJMH 26 | 41-50 | Non Manager | Doctor (Specialist) | Male |
| EJMH 27 | 51-63 | Non Manager | Doctor (Specialist) | Female |
| EJMH 28 | 51-63 | Non Manager | Doctor (Specialist) | Male |
| EJMH 29 | 51-63 | Non Manager | Doctor (General Practictionner) | Male |
| EJMH 30 | 31-40 | Non Manager | Doctor (Specialist) | Male |
| EJMH 31 | 51-63 | Non Manager | Doctor (Specialist) | Male |
| EJMH 32 | 51-63 | Operational Manager | Radiology technician | Female |
| EJMH 33 | 20-30 | Non Manager | Nurse | Female |
| EJMH 34 | 20-30 | Non Manager | MidWife | Female |
| EJMH 35 | 51-63 | Non Manager | Laboratory technician | Female |
| EJMH 36 | 31-40 | Non Manager | Nurse anthesiologist | Female |
| EJMH 37 | 51-63 | Non Manager | Auxillary Nurse | Female |
| EJMH 38 | 31-40 | Non Manager | Doctor (General Practictionner) | Female |
| EJMH 39 | 31-40 | Non Manager | Nurse | Male |
| EJMH 40 | 51-63 | Operational Manager | Nurse | Male |
| EJMH 41 | 51-63 | Non Manager | Nurse | Male |
| EJMH 42 | 51-63 | Intermediate Manager | Doctor (General Practictionner) | Male |
| EJMH 43 | 51-63 | Senior Manager | Administrator | Female |
