# Supplementary material for: Does public service motivation matter in Moroccan public hospitals? A multiple embedded case study
Source: Int J Equity Health. 2019 Oct 22;18:160. doi: 10.1186/s12939-019-1053-8 (PMC6805632; doi:10.1186/s12939-019-1053-8)
Supplement: Supplementary file 5 — Additional file 5: Sociodemographic characteristics, case study 3 (RKMH). [file 12939_2019_1053_MOESM5_ESM.docx]

Additional file 5: Sociodemographic characteristics of respondants from case study 3

| Code | Age | Managerial function | Professional profile | Genre |
| --- | --- | --- | --- | --- |
| RKMH 1 | 41-50 | Non Manager | Doctor (General Practictionner) | Male |
| RKMH 2 | 41-50 | Intermediate Manager | Pharmacist | Male |
| RKMH 3 | 41-50 | Non Manager | Doctor (Specialist) | Female |
| RKMH 4 | 51-63 | Operational Manager | Nurse | Female |
| RKMH 5 | 41-50 | Operational Manager | Administrator | Male |
| RKMH 6 | 51-63 | Senior Manager | Nurse | Male |
| RKMH 7 | 51-63 | Operational Manager | Nurse | Male |
| RKMH 8 | 51-63 | Operational Manager | Technician (Technical staff) | Female |
| RKMH 9 | 41-50 | Non Manager | Cashier (Technical staff) | Male |
| RKMH 10 | 31-40 | Non Manager | Nurse | Male |
| RKMH 11 | 20-30 | Non Manager | Nurse | Female |
| RKMH 12 | 20-30 | Non Manager | Nurse anthesiologist | Female |
| RKMH 13 | 20-30 | Non Manager | Radiology technician | Female |
| RKMH 14 | 31-40 | Non Manager | MidWife | Female |
| RKMH 15 | 51-63 | Senior Manager | Administrator | Male |
| RKMH 16 | 41-50 | Non Manager | Doctor (Specialist) | Female |
| RKMH 17 | 20-30 | Non Manager | Radiology technician | Female |
| RKMH 18 | 20-30 | Non Manager | Nurse anthesiologist | Male |
| RKMH 19 | 31-40 | Non Manager | Nurse | Male |
| RKMH 20 | 51-63 | Non Manager | Nurse | Female |
| RKMH 21 | 31-40 | Non Manager | MidWife | Female |
| RKMH 22 | 31-40 | Non Manager | technician (Technical staff) | Male |
| RKMH 23 | 41-50 | Senior Manager | Doctor (General Practictionner) | Male |
| RKMH 24 | 31-40 | Operational Manager | Nurse | Male |
| RKMH 25 | 41-50 | Non Manager | Technician (Technical staff) | Male |
| RKMH 26 | 41-50 | Non Manager | technician (Technical staff) | Male |
