# Supplementary material for: Does public service motivation matter in Moroccan public hospitals? A multiple embedded case study
Source: Int J Equity Health. 2019 Oct 22;18:160. doi: 10.1186/s12939-019-1053-8 (PMC6805632; doi:10.1186/s12939-019-1053-8)
Supplement: Supplementary file 6 — Additional file 6: Sociodemographic characteristics, case study 4 (SMBA). [file 12939_2019_1053_MOESM6_ESM.docx]

Additional file 6: Socio demographic characteristics from case study 4

| Code | Age | Managerial function | Professional profile | Genre |
| --- | --- | --- | --- | --- |
| SMBA 1 | 51-63 | Non Manager | Nurse | Male |
| SMBA 2 | 31-40 | Non Manager | Nurse | Female |
| SMBA 3 | 41-50 | Senior Manager | Doctor (General Practictionner) | Male |
| SMBA 4 | 31-40 | Senior Manager | Nurse (Filling administrative position) | Male |
| SMBA 5 | 51-63 | Senior Manager | Nurse | Male |
| SMBA 6 | 41-50 | Line Manager | MIdWife | Female |
| SMBA 7 | 51-63 | Non Manager | Nurse | Female |
| SMBA 8 | 51-63 | Non Manager | Nurse | Female |
| SMBA 9 | 51-63 | Non Manager | Nurse | Female |
| SMBA 10 | 51-63 | Non Manager | Nurse | Female |
| SMBA 11 | 20-30 | Non Manager | MIdWife | Female |
| SMBA 12 | 31-40 | Non Manager | Nurse anthesiologist | Female |
| SMBA 13 | 31-40 | Non Manager | Nurse | Female |
| SMBA 14 | 31-40 | Non Manager | Doctor (General Practictionner) | Female |
| SMBA 15 | 31-40 | Non Manager | Doctor (Specialist) | Female |
| SMBA 16 | 41-50 | Non Manager | Doctor (General Practictionner) | Male |
| SMBA 17 | 51-63 | Line Manager | Nurse | Male |
| SMBA 18 | 41-50 | Line Manager | Doctor (General Practictionner) | Male |
| SMBA 19 | 41-50 | Non Manager | Doctor (Specialist) | Female |
| SMBA 20 | 41-50 | Non Manager | Doctor (General Practictionner) | Male |
| SMBA 21 | 51-63 | Non Manager | Technician (Technical staff) | Male |
| SMBA 22 | 41-50 | Non Manager | Technician (Technical staff) | Male |
| SMBA 23 | 51-63 | Line Manager | Doctor (General Practictionner) | Male |
| SMBA 24 | 51-63 | Line Manager | Nurse | Female |
| SMBA 25 | 31-40 | Operational Manager | Nurse | Male |
| SMBA 26 | 31-40 | Non Manager | technician (Technical staff) | Male |
| SMBA 27 | 41-50 | Non Manager | Technician (Technical staff) | Female |
| SMBA 28 | 41-50 | Non Manager | Administrator | Male |
| SMBA 29 | 31-40 | Non Manager | Technician (Technical staff) | Male |
| SMBA 30 | 31-40 | Non Manager | Cashier (Technical staff) | Female |
| SMBA 31 | 51-63 | Non Manager | technician (Technical staff) | Female |
| SMBA 32 | 41-50 | Senior Manager | Doctor (Specialist) | Male |
| SMBA 33 | 31-40 | Non Manager | Pharmacist | Female |
| SMBA 34 | 20-30 | Non Manager | Pharmacy technician | Female |
| SMBA 35 | 51-63 | Non Manager | Nurse | Female |
| SMBA 36 | 41-50 | Operational Manager | Administrator | Male |
| SMBA 37 | 51-63 | Non Manager | Nurse (Operating theator) | Female |
| SMBA 38 | 31-40 | Non Manager | Nurse | Female |
| SMBA 39 | 31-40 | Non Manager | Nurse | Male |
| SMBA 40 | 31-40 | Operational Manager | Doctor (Specialist) | Male |
| SMBA 41 | 20-30 | Non Manager | Psychiatric nurse | Male |
| SMBA 42 | 31-40 | Non Manager | Doctor (Specialist) | Female |
| SMBA 43 | 31-40 | Non Manager | Doctor (Specialist) | Female |
| SMBA 44 | 31-40 | Non Manager | Doctor (General Practictionner) | Female |
| SMBA 45 | 51-63 | Non Manager | Doctor (General Practictionner) | Male |
